# Supplementary material for: Implementation of a goal-directed Care Bundle for intracerebral hemorrhage: Results of embedded process evaluation in the INTERACT3 trial
Source: PLOS Glob Public Health. 2024 Dec 19;4(12):e0003711. doi: 10.1371/journal.pgph.0003711 (PMC11658503; doi:10.1371/journal.pgph.0003711)
Supplement: S1 Table — (DOCX) [file pgph.0003711.s001.docx]

**S1 Table. Percentage of participating sites for survey and interviews by country**

| **Country** | **N (%) participating sites for survey** | **N (%) participating sites for interview** | **Total participating sites in the INTERACT3 trial** |
| --- | --- | --- | --- |
| Brazil | 2 (40.0) | 0 (0.0) | 5 |
| India | 5 (83.3) | 2 (33.3) | 6 |
| Nigeria | 3 (100.0) | 1 (33.3) | 3 |
| Pakistan | 3 (100.0) | 2 (66.7) | 3 |
| Sri Lanka | 3 (42.9) | 1 (14.3) | 7 |
| Vietnam | 3 (50.0) | 2 (33.3) | 6 |
| Chile | 5 (71.4) | 1 (14.3) | 7 |
| Peru | 1 (100.0) | 1 (100.0) | 1 |
| Mexico | 0 (0.0) | 1 (100.0) | 1 |
| **Total** | **25** | **11** | **39** |
