# Supplementary material for: Implementation of a goal-directed Care Bundle for intracerebral hemorrhage: Results of embedded process evaluation in the INTERACT3 trial
Source: PLOS Glob Public Health. 2024 Dec 19;4(12):e0003711. doi: 10.1371/journal.pgph.0003711 (PMC11658503; doi:10.1371/journal.pgph.0003711)
Supplement: S2 Table — (DOCX) [file pgph.0003711.s002.docx]

**S2 Table. Characteristics of the purposive sampled interview participants**

| Hospital | No. | Country | Hospital level | Department | Doctor (n) | Nurse (n) | Patient / Carer (n) | Rate of recruitment* | Data entry quality† |
| --- | --- | --- | --- | --- | --- | --- | --- | --- | --- |
| 2001 | 1 | Peru | Tertiary | Neurology | 2 (principle doctor/attending specialist) | 0 | 1 | 83% | 1 |
| 2008 | 2 | Chile | Tertiary | Neurology | 1 (attending specialist) | 0 | 2 | 14% | 1 |
| 4002 | 3 | Vietnam | Tertiary | Neurology | 2 (1 principle doctor, 1 attending specialist) | 0 | 0 | 25% | 1 |
| 4014 | 4 | Vietnam | Tertiary | Neurology | 1 (attending specialist) | 0 | 1 | 237% | 1 |
| 5001 | 5 | Pakistan | Tertiary | Medicine | 2 (neurology residents) | 0 | 1 | 31% | 2 |
| 5003 | 6 | Pakistan | Tertiary | Neurology | 1 (neurology resident) | 2 | 0 | 18% | 1 |
| 8001 | 7 | Nigeria | Tertiary | Medicine | 2 (neurology resident) | 1 | 2 | 39% | 2 |
| 9102 | 8 | India | Tertiary | Neurology | 1 (neurologist) | 0 | 0 | 22% | 1 |
| 9106 | 9 | India | Tertiary | Neurology | 1 (neurologist) | 0 | 0 | 132% | 1 |
| 9207 | 10 | Sri Lanka | Tertiary | Neurology | 2 (1 consultant neurologist, 1 neurology resident) | 0 | 0 | 62% | 2 |
| 6001 | 11 | Mexico | Tertiary | Stroke clinic | 1 (neurology resident) | 0 | 1 | 8% | 1 |
| **Total** | **27** |  |  |  | **16** | **3** | **8** |  |  |

BP denotes blood pressure, SBP systolic blood pressure

*Rate of recruitment calculated according to actual enrolment number divided by expected weekly enrolment. A low rate of recruitment is defined by less than 50% of expected enrolment.

†Data entry quality evaluated according to routine monitoring data, field notes and monthly implementation performance reports extracted from case report forms with scale 1 indicates good, 2 indicates moderate, and 3 indicates poor.
