# Supplementary material for: Implementation of a goal-directed Care Bundle for intracerebral hemorrhage: Results of embedded process evaluation in the INTERACT3 trial
Source: PLOS Glob Public Health. 2024 Dec 19;4(12):e0003711. doi: 10.1371/journal.pgph.0003711 (PMC11658503; doi:10.1371/journal.pgph.0003711)
Supplement: S3 Table — (DOCX) [file pgph.0003711.s003.docx]

**S3 Table. Reason of exclusion by country in screened participants**

| **Screening** | **Brazil** | **Chile** | **India** | **Mexico** | **Nigeria** | **Pakistan** | **Peru** | **Sri Lanka** | **Vietnam** | **China** | **Total** |
| --- | --- | --- | --- | --- | --- | --- | --- | --- | --- | --- | --- |
| Screened | 164 | 214 | 100 | 59 | 141 | 119 | 39 | 330 | 298 | 9374 | 10838 |
| *Exclusion* | 128 (78.0%) | 166 (77.6%) | 33 (33.0%) | 50 (84.7%) | 71 (50.4%) | 70 (58.8%) | 27 (69.2%) | 96 (29.1%) | 143 (48.0%) | 3018 (32.2%) | 3802 (35.1%) |
| Presentation>6 h from the onset of symptoms | 68 (53.1%) | 122 (73.5%) | 22 (66.7%) | 35 (70.0%) | 71 (100.0%) | 47 (67.1%) | 23 (85.2%) | 78 (81.3%) | 99 (69.2%) | 2000 (66.3%) | 2565 (67.5%) |
| No consent | 33 (25.8%) | 26 (15.7%) | 9 (27.3%) | 2 | 0 | 19 | 2 | 12 (12.5%) | 39 (27.2%) | 517 (17.2%) | 659 (17.3%) |
|  |  |  |  | (4.0%) | (0.0%) | (27.1%) | (7.4%) |  |  |  |  |
| Other reasons | 27 | 18 | 2 | 13 | 0 | 4 | 2 | 6 | 5 | 489 | 572 (15.0%) |
|  | (0.0%) | (0.0%) | (6.1%) | (26.0%) | (0.0%) | (5.7%) | (7.4%) | (4.2%) | (3.5%) | (16.3%) |  |
