# Supplementary material for: Implementation of a goal-directed Care Bundle for intracerebral hemorrhage: Results of embedded process evaluation in the INTERACT3 trial
Source: PLOS Glob Public Health. 2024 Dec 19;4(12):e0003711. doi: 10.1371/journal.pgph.0003711 (PMC11658503; doi:10.1371/journal.pgph.0003711)
Supplement: S4 Table — (DOCX) [file pgph.0003711.s004.docx]

**S4 Table. Reach and dose of patients for the care bundle implementation**

| **Care bundle** | **Total**  **N=3221** |
| --- | --- |
| **SBP ≥140 mmHg** | 2905/3221 (90.2%) |
| Intensive BP lowering treatment during the first 24 hrs | 2542/3221 (78.9%) |
| SBP target <140mmHg reached* | 2809/2905 (96.7%) |
| **Non-diabetic patients BGL>7.8 mmol/L or Diabetic patients BGL>10.0 mmol/L** | 1094/3175 (34.5%) |
| Treatment for glycemia control during the first 24 hrs | 250/3221 (7.8%) |
| Blood glucose target of 6.1–7.8 mmol/L in patients without diabetes and 7.8–10.0 mmol/L in patients with diabetes reached† | 147/1094 (13.4%) |
| **Body Temperature > 37.5°C** | 52/3214 (1.6%) |
| Treatment for pyrexia during the first 24 hrs | 3140/3219 (97.5%) |
| Temperature target (37.5°C) reached† | 45/52 (86.5%) |
| **International normalised ratio ≥1.5** | 25/3113 (0.8%) |
| International normalised ratio target <1.5 reached‡ | 12/25 (48.0%) |

*Reached by day 7

†Reached by day 3

‡Reach by first 24 hrs
