# Supplementary material for: Implementation of a goal-directed Care Bundle for intracerebral hemorrhage: Results of embedded process evaluation in the INTERACT3 trial
Source: PLOS Glob Public Health. 2024 Dec 19;4(12):e0003711. doi: 10.1371/journal.pgph.0003711 (PMC11658503; doi:10.1371/journal.pgph.0003711)
Supplement: S5 Table — (DOCX) [file pgph.0003711.s005.docx]

**S5 Table. Treatment changes for ICH management by country from the survey results**

| **Item** | **Total**  **n=47** | **India**  **n=12** | **Nigeria**  **n=8** | **Pakistan**  **n=6** | **Chile/Peru**  **n=7** | **Sri Lanka**  **n=6** | **Vietnam**  **n=8** |
| --- | --- | --- | --- | --- | --- | --- | --- |
| **Treatment and management changed after participating in INTERACT3** | 33 (70.2%) | 6 (50.0%) | 7 (87.5%) | 6 (100.0%) | 3 (42.9%) | 4 (66.7%) | 7 (87.5%) |
| **Part of change** |  |  |  |  |  |  |  |
| Blood pressure control | 31 (93.9%) | 5 (83.3%) | 7 (100.0%) | 6 (100.0%) | 3 (100.0%) | 4 (100.0%) | 6 (85.7%) |
| Glycemic control | 20 (60.6%) | 4 (66.7%) | 4 (57.1%) | 6 (100.0%) | 3 (100.0%) | 3 (75.0%) | 5 (71.4%) |
| Body temperature control | 17 (51.5%) | 4 (66.7%) | 4 (57.1%) | 3 (50.0%) | 1 (33.3%) | 3 (75.0%) | 6 (85.7%) |
| Anticoagulation reversal | 8 (24.2%) | 1 (1.7%) | 0 (0.0%) | 1 (16.7%) | 0 (0.0%) | 1 (25.0%) | 5 (71.4%) |
| Others | 2 (6.1%) | 1 (1.7%) | 0 (0.0%) | 0 (0.0%) | 1 (33.3%) | 0 (0.0%) | 0 (0.0%) |

n = number of survey respondents
