# Supplementary material for: Implementation of a goal-directed Care Bundle for intracerebral hemorrhage: Results of embedded process evaluation in the INTERACT3 trial
Source: PLOS Glob Public Health. 2024 Dec 19;4(12):e0003711. doi: 10.1371/journal.pgph.0003711 (PMC11658503; doi:10.1371/journal.pgph.0003711)
Supplement: S6 Table — (DOCX) [file pgph.0003711.s006.docx]

**S6 Table. Embedding the Care Bundle into routine care by normalisation process theory (NPT) domains**

| **NPT domains** | **Explanation** | **Quote** |
| --- | --- | --- |
| ***Coherence*** | Good understand of the study rationale | *“To ascertain the benefits of early and intense BP reduction in ICH. Although it is known that BP control is beneficial in ICH, when provided as part of an acute care bundle along with control of other vital parameters like sugar, INR and temperature, the benefit could be more. I had no concerns as the aspects are known to be beneficial / not harmful.” (No.9, Sri Lanka, neurology resident)* |
|  | Usual care is easier/simpler since the care bundle is intensive and strict | *“Easier to carry out what we commonly use in our hospital, which is a control, a less strict blood pressure goal and a glycemic monitoring every 6 or 8 ours. It is not so strict in that sense.” (No. 10, Mexico, neurology resident)*  *“Routine care was easier, because INTERACT bundle requires intense monitoring. Management otherwise is more or less the same but these time targets are not met in routine care.” (No. 9, Sri Lanka, Neurology resident)* |
|  | Appreciate the care bundle and perceive a benefit in participating the trial | *“It seems to me that the study is useful, it seems important to me to know well what is the best treatment that we can give to our patients”* (N°2; Chile, neurology resident)  *“Theoretically, the intervention is similar in terms of the rigor of control and strict management and we hope that the hospital will reach that level of rigor*.” (N°2; Chile, neurology resident)  *“because of the training, while in school we don’t use this things, it makes us here to use the infusion pump in the ward and even to know it because we are seeing it for the first time and it makes us to take care of our patients easily...the sugar level is high; we use insulin supplied for this INTERACT 3” (Nigeria, nurse)* |
| ***Cognitive participation*** | In the context of limited resources, conduct the trial made the hospital possible to have equipment medications for patient care | *“This INTERACT3 helped in monitoring of patients, it made us to have monitor machine, infusion pump machine and many other things supplied... Patients that are captured for this study but we even used it for other patients like monitor…That of interact 3 is easier because many of the patients will come and they will not have money to buy drugs. They will not even have money to do CT scan. With this INTERACT 3, it made us even to take care of them easily and they respond very well.” (No.6, Nigeria, Nurse)* |
|  | Clinicians perceived the benefits of care bundle to ICH patients | *“I believe it will benefit the patients, as we are not only monitoring the blood pressure, instead we are observing other parameters as mentioned earlier. Obviously, as you know, it is important to control blood pressure, similarly it is important to control sugar and to control INR, if it above 1.5 we have to manage the patients accordingly. Or if a patient is hyperthermic then monitoring all parameters will help manage the patient accordingly.” (No.4, Pakistan, neurology resident)* |
|  | Increased workload for intensive monitoring influence the commitment | *“Cross-over was tough, the other healthcare staff weren’t very happy to take additional patients who required intense monitoring” (No. Sri Lanka 002, doctor)*  *“Other issues are staff related, as discussed earlier they are busy, and they are overloaded as a result -they do not pay attention to the trial” (Pakistan, No. _implementer)* |
| ***Collective action*** | It was important to develop proper pathway, adapt treatment protocols and sufficient learning materials to support the implementation of the intervention in practice | *“We can implement these things in our hospital very easily, but at the very beginning we have to develop a guideline and the proper protocol and that will require a lot of efforts. But once we will develop these things that will be very easily. We have stroke code as well as a proper pathways regarding it. “ (No.5, Pakistan, Nurse)*  *“We have to sticked to the protocol. We start oral antihypertensive parallel to IV medication so that the IV infusion can be tracked down quickly.” (No.9, Sri Lanka, Neurology resident)*  *“Obviously, initially we had to face a lot of challenges, for which we coordinated with PI and also contacted the coordinator. Eventually the issues were settled. We were given the forms and other reading materials (such as the cards of INTERACT3 trial), so these reading materials were a lot easier to understand.” (No. 4, Pakistan, neurology resident)*  *“The subject has been the management of blood pressure (BP). The BP could not be treated with the doses proposed with the protocol…. And we had some problems in the implementation, but all these problems had been solved little by little” (N°1; Chile, neurology resident)*  “*The management of hypertension is what has cost the most, it has cost a lot. It has been difficult because we have had two extremes: some patients in whom we have not had to perform many interventions and in others it has been difficult for us to reach the desired range”* *(N°1; Chile, neurology resident).* |
|  | Multiple departments collaboration and multidisciplinary care to support the implementation of care bundle | *“We informed the neurology team on an urgent basis to review patients. A proper management plan is then started…I would say over this we have seen an incredible team display from both the emergency as well as neurology team we have been able to identify the patients early to ensure effective timely management of the patients.” (No.5, Pakistan, Neurology resident)*  *“Same is the case of blood glucose levels, we have queries as how long do we have to continue insulin or when to stop insulin infusion during the trial. We have to consult the specialty physician like internal medicine, endocrinology and their suggestive treatment.” (No. 4, Pakistan, neurology resident)*  “*One of the main problems is in urgency. Patients spend a lot of time in the emergency room (at least 12 hours) and it has been difficult to approach interventions, for example with the correction of coagulation” (N° 2 Chile, neurology resident)*  *“The protocol has been slower than expected; several patients have not entered the protocol for different reasons and we have had difficulties in the registration, mainly in the emergency” (N° 2 Chile, neurology resident)* |
| ***Reflexive monitoring*** | Constant and regular basis trainings are helpful to maintain the care bundle implementation, particularly in ED | *“Because the staff training is more in this department and we need a training and development to develop the things as well and to maintain this guideline in the department. We need regular basis of the sessions on the things to develop the awareness in the staff as well.” (No.5, Pakistan, Nurse)*  *“So you know they do not communicate their concerns at such points and I think the ER Staff needs to be trained a bit more about the complexity of the protocol; though our staff is well trained on the topic and its importance.” (No.4, Pakistan, Neurology resident)*  *“The training is good enough but depending on the duration of the study, there will be need for retraining at certain, especially when newer members of the team are joining the research process there will be need for training and retraining.” (No.6, Nigeria, Neurology resident)* |
|  | Elements include detailed guidance and policy are important for integration and sustainability | *“I believe if we develop a proper policy or a guideline for the implementation of this plan on a larger scale, then obviously everyone has to follow it.” (No.4, Pakistan, Neurology resident)*  *“If we proper develop the pathways and we implement them in the hospital or across the hospital so it will be easy and we will give a proper training to the staff at the grass root level because the turnover of the staff is very high so it will be easy.” (No.5, Pakistan, Nurse)*  *“So that if you can provide good quality or if we aim to provide good quality care then we should ensure that all of the equipment should be available, proper monitoring of the four parameters that we say our central to INTERACT 3. So that would be a real risk and needs to be accessed early.” (No.5, Pakistan, Neurology resident)* |
|  | Increase prehospital awareness, patient and community symptoms identification | *“We should increase awareness of stroke in the community first …The pre-notification for the emergency nursing staff to get ready to receive the stroke patients without delay.” (India_002)*  *“Challenges concerning our patients because of our community, most of our patients when they* have stroke instead of bringing them to the hospital some will go to the herbalist, our community because they did not believe it is God’s wish, they will believe it is witch or wizard, so they won’t come to the hospital immediately.*” (Nigeria, nurse)*  *“Patients who have presented a parenquimathosis hemorrhage do not always appear within the adequate time, the delay in his arrival at the Emergency Service is the main problem.” (Mexico, Dr)* |
|  | Recognised the importance of adopted into routine care and widely used, but factors such as resources, knowledge and admit of the benefits on the care bundle need to be solved to ensure the sustainability | *“I think some hospitals might not have the neuro-imaging techniques necessary to make the diagnosis.” (Nigeria, Dr 1)*  *“One is facilities. Like, I am not sure if most hospitals have infusion pumps in their medical emergencies. So we have to provide these amenities.” (Nigeria, Dr 2)*  *“Hospitals that do not have nicardipine or equipment or drugs to manage blood glucose, will not be able to follow the study protocol. Room conditions might not be adequate.” (Vietnam, Neurologist)* |

BGL denotes blood glucose level, BP blood pressure, ED emergency department, ICH intracerebral hemorrhage, PI principal investigator
