# Supplementary figures and images for: Implementation of a goal-directed Care Bundle for intracerebral hemorrhage: Results of embedded process evaluation in the INTERACT3 trial
Source: PLOS Glob Public Health. 2024 Dec 19;4(12):e0003711. doi: 10.1371/journal.pgph.0003711 (PMC11658503; doi:10.1371/journal.pgph.0003711)

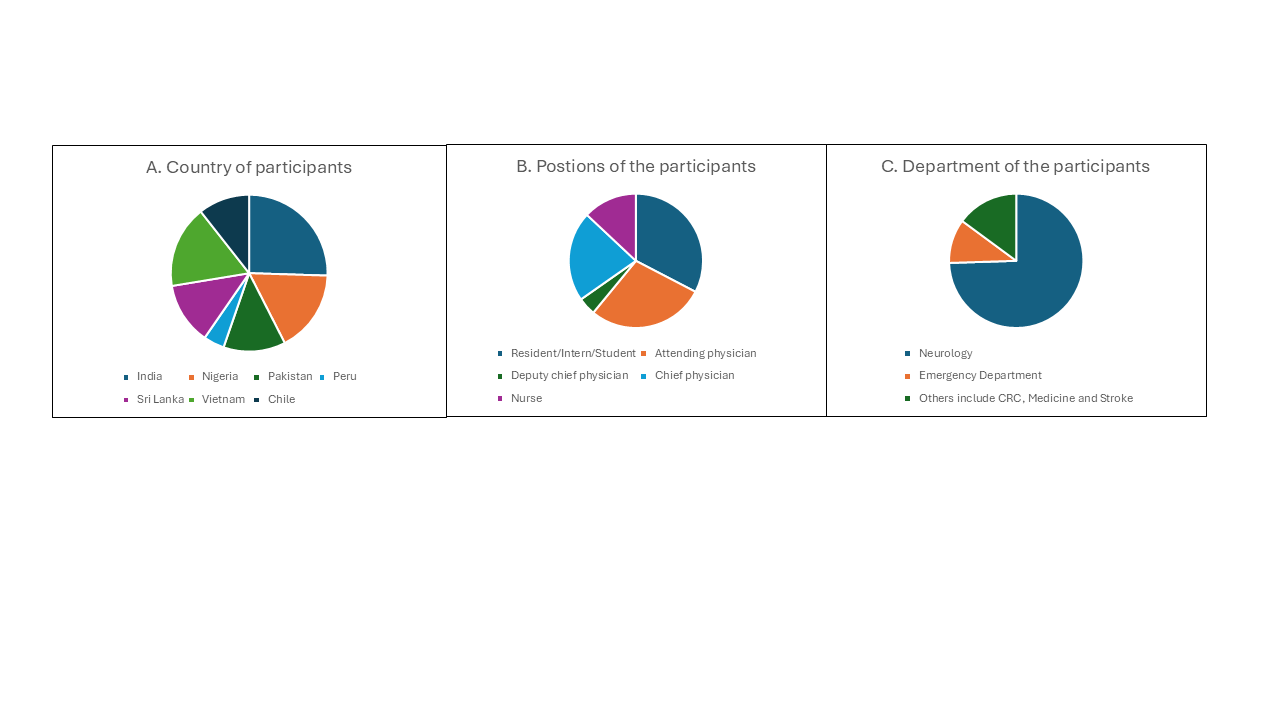

Supplement: S1 Fig — (TIF) [file pgph.0003711.s007.TIF]

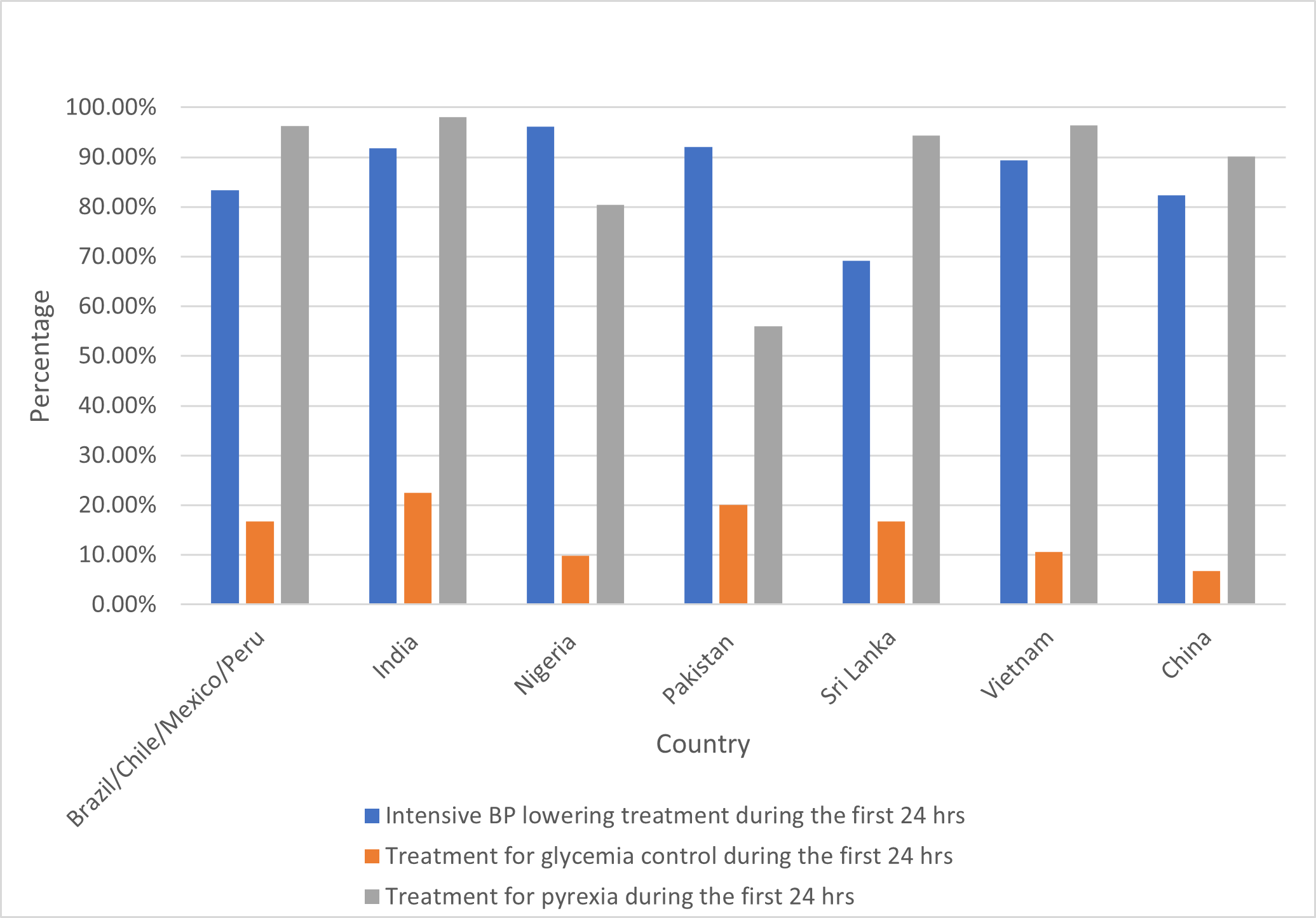

Supplement: S2 Fig — (TIF) [file pgph.0003711.s008.tif]

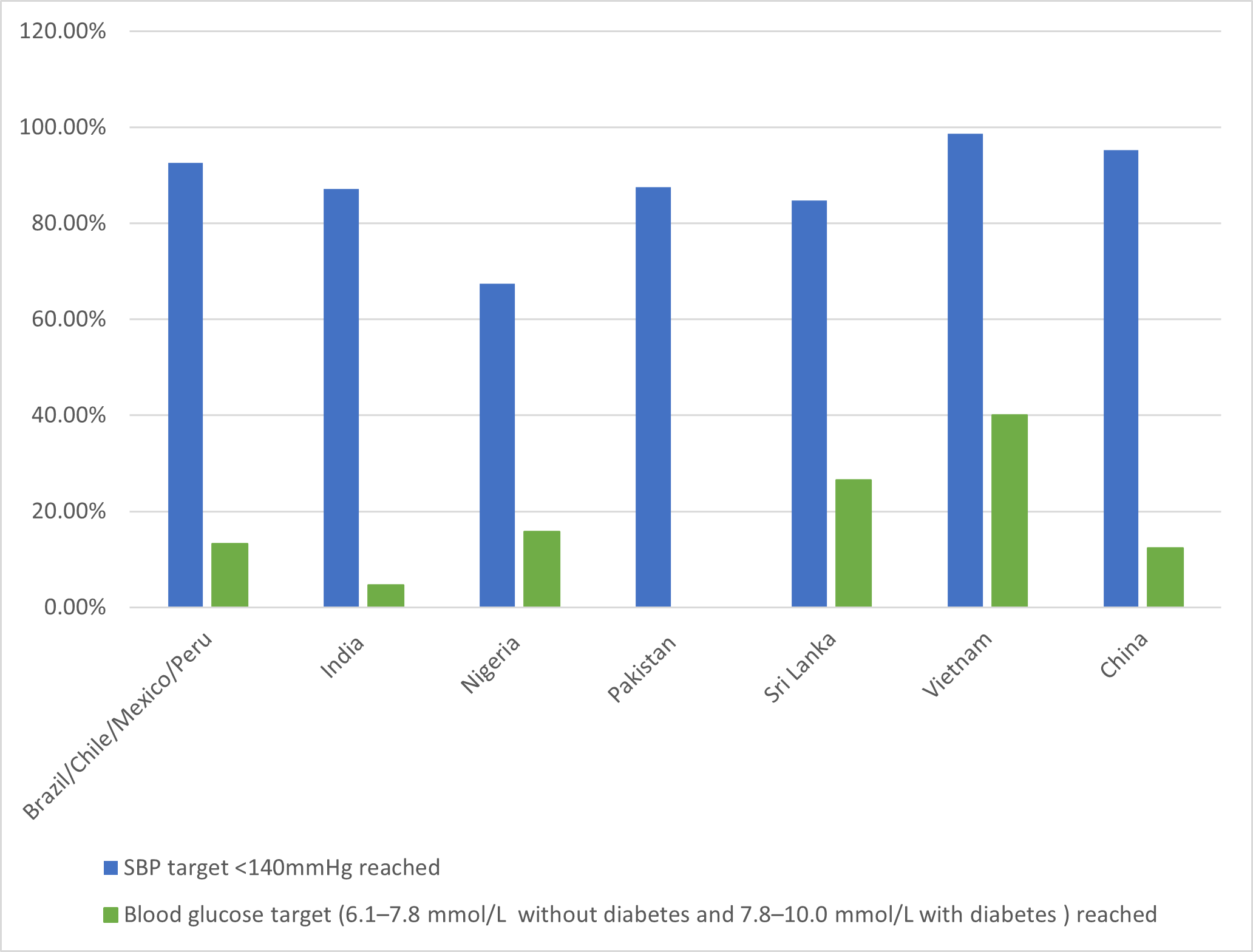

Supplement: S3 Fig — (PNG) [file pgph.0003711.s009.png]

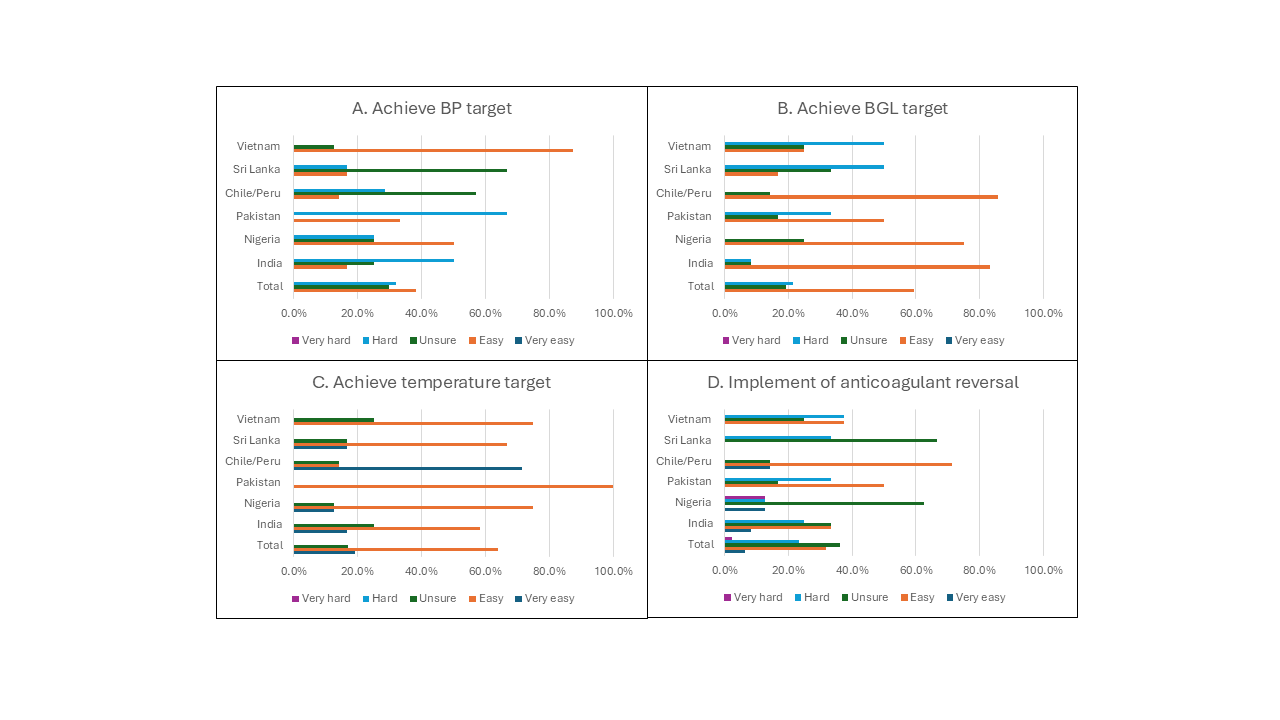

Supplement: S4 Fig — (TIF) [file pgph.0003711.s010.TIF]

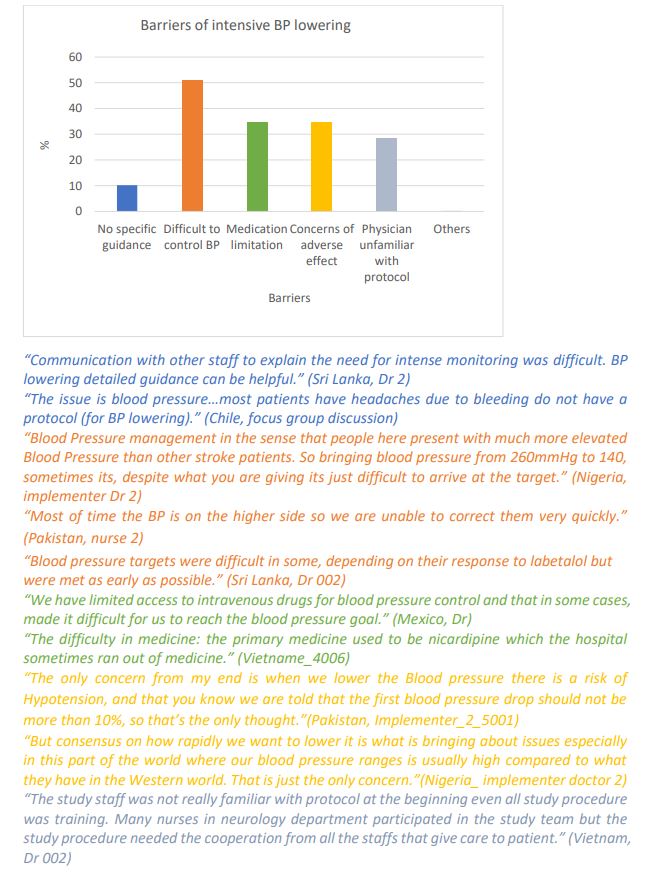

Supplement: S5 Fig — (JPG) [file pgph.0003711.s011.JPG]

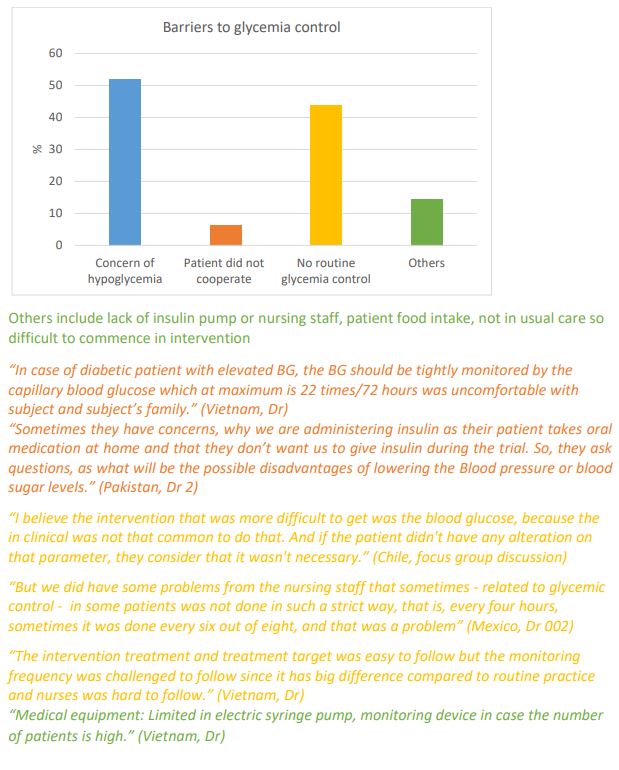

Supplement: S6 Fig — (JPG) [file pgph.0003711.s012.JPG]
