# Supplementary material for: Implementation of a goal-directed Care Bundle for intracerebral hemorrhage: Results of embedded process evaluation in the INTERACT3 trial
Source: PLOS Glob Public Health. 2024 Dec 19;4(12):e0003711. doi: 10.1371/journal.pgph.0003711 (PMC11658503; doi:10.1371/journal.pgph.0003711)
Supplement: Attachment — Submitted filename: Responses to Reviewer_PLOS GPH_clean.docx [file pgph.0003711.s014.docx]

19 August 2024

Editorial Office

PLOS Global Public Health

Dear Dr Buna Bhandari,

**Re: PGPH-D-24-01251– “Implementation of a goal-directed care bundle for intracerebral hemorrhage: results of embedded process evaluation in the INTERACT3 trial”**

We appreciate the comments and suggestions from Editors and Reviewers. We have responded to each of these issues point by point, as outlined below, and made highlighted changes in a revision of the manuscript.

**Reviewer #1: The manuscript meets all the requirements of the Journal, is well-written, interesting and contains the necessary elements for the reproduction of the research undertaken by the authors. An additional advantage of the work is its applied purpose.
I recommend publication in its present form.**

Response: Thanks for your positive comments.

**Reviewer #2: Thank you for the opportunity to review this manuscript reporting on a process evaluation embedded in a stepped-wedge, cluster-randomized, Intensive Care Bundle with Blood Pressure Reduction in Acute Cerebral Hemorrhage Trial (INTERACT3). The reported process evaluation was conducted in 7 LMIC. A total of 27 interviews (16 doctors, 3 nurses, and 8 patients/carers) and 3 focus group discussions were conducted, alongside 47 responses from the quality control survey at 25 hospitals. The manuscript is well written and presents relevant findings. However, I believe that there are a number of points that should be addressed by the authors.

1. The introduction is very light on details regarding ICH and background information on why this multifaceted care bundle was selected. I would appreciate some more literature and context.**

Response: Thanks for the suggestion. We added more information regarding the background and rationale of the study: “Intracerebral hemorrhage (ICH) is the most serious and least treatable form of stroke, accounting for approximately 20% of the 20 million new strokes that occur in the world each year.^1^ However, the burden of ICH is the greatest in low- and middle-income countries (LMIC) where the incidence is very high from the high prevalence of hypertension, unhealthy diets and other risk factors,^1^ and there is limited access to surgical or medical treatments.^2^ Hematoma expansion (HE) is a strong predictor of disability and mortality after ICH, with every increase of 1 ml in absolute haematoma volume, it leads to a 7% greater likelihood for patients to shift from independence to disability.^3^ HE occurs mostly within 3 hrs from the time of ICH onset and stops progressing after 24 hrs.^4^ Therefore, timely control of HE is an important therapeutic target in ICH management. Since blood pressure, glycaemia and temperature are often altered from impaired autoregulation after ICH and are associated with haematoma expansion, death and disability, the active management for ICH should target on early management on these physiological parameters.

Care bundles are a set of evidence-informed interventions performed collectively and reliably to improve the quality of care. The single-centre before-after Acute Bundle of Care for Intracerebral Haemorrhage (ABC-ICH) was the first study that has shown combined interventions including anticoagulation reversal and BP control, and immediate access to neurosurgical and critical care services reduced 10.8% of the case fatality at 30 days.^5^ Our third Intensive Care Bundle with Blood Pressure Reduction in Acute Cerebral Hemorrhage Trial (INTERACT3) has extended the knowledge of the benefits of bundled care, which was specifically undertaken to determine if a simple, multi-faceted, Care Bundle incorporating time- and target-based protocols for the management of abnormal physiological variables could improve outcomes for patients with ICH in a broad range of health care settings.^6,7^ ”. **2. The major limitation of the paper is the focus on a range of countries (7 to 10 - this is somewhat unclear, see my next comment) for the process evaluation. The authors previously published the process evaluation conducted in China - why were all of the other countries grouped together? What is the rationale? What are the implications of combining all of the data across countries in a single paper? This raises other issues discussed below.**

Response: The INTERACT3 trial was conducted in China as a vanguard phase from November 2017 to November 2020, while all the sites outside of China occurred between March 2019 and March 2021. Nearly 85% of the participants were from China and 82 out of the total 122 participating hospitals were in China. Considering the China was mostly conducted before the other countries in the vanguard phase and included the majority of the participants were from China, and the findings from the vanguard phase could inform the implementation in the other countries, we reported the findings separately in the previous publication. We have added the rationale and justification in the Methods: “Since the majority of the participants and the hospitals involved in the INTERACT3 trial were from China in the vanguard phase, we conducted and reported the process evaluation separately by China vs. other countries.”

**3. How many countries were included in the process evaluation? At one point, it is mentioned that "A quality control survey was collected to understand the experience and project operational barriers to implementing the intervention at sites in Brazil, Chile, India, Mexico, Nigeria, Pakistan, Peru, Sri Lanka, and Vietnam" whereas later it is mentioned "11 sites agreed to participate from India, Sri Lanka, Vietnam, Pakistan, Chile, Nigeria and Mexico." How many countries were included in this process evaluation, and how many sites across these countries (how many per country?)? This is also complicated by the fact that data is present from countries that should be absent from this paper (e.g., China data in Table S2 - Supposed to be in its own paper and already published).**

Response: We used mixed-methods approach including survey, interview and focus group discussion to collect data for process evaluation. The number and percentage of the participating sites for survey and interview per country were added in the Table S1. The participating sites responses rates by each country ranged from 40% to 100%, however, there was no response from Mexico because we had only one site involved in INTERACT3. We added to the limitation: “Although we made efforts to include all the participating sites to complete the survey, we acknowledge the limitation that some of the countries had no or very few sites to participate the survey, which might cause volunteer bias.”

Since the process evaluation for China was published before the trial completion, the data that related to the enrolment (Table S2) and care bundle delivery (Figure S2 and S3) could not be provided. Therefore, we included these data in the current study as a supplement to compare across the countries. **4. How was the interview guide elaborated? Was it based on a theoretical model? What were the questions? What the interview guide pilot tested? The interview guide should be attached as an additional file.**

Response: To elaborate, we added “The process evaluation design was based on the UK Medical Research Council (MRC) process evaluation guidance and Normalization Process Theory (NPT). Interview guide questions were informed by the normalization process theory domains to enable an in-depth understanding of the integration of the care bundle into routine practice.” The data collection materials including interview guide, survey questions and focus group discussion guide (are attached as supplementary files) were tested with 2 clinicians from the regional coordinating center from Chile and China. These data collection materials were published in the previous study of process evaluation for China (Ouyang M, et al. CED. 2021 Dec 20:1-11. doi: 10.1159/000520669), and we have attached it as a supplementary file.

**5. While Table 1 is interesting, there are a number of issues. First, some implementation outcomes are grouped together (acceptability and appropriateness). While I understand that these can be conceived as being similar, I believe there would be value in separating these out like the other outcomes. Second, and this is fundamental, there is a need to present the results for each implementation outcome for each country. There is little value in present an overall general theme and some quotes for some of the countries, given that all countries would have widely different contexts. Consequently, the authors should re-work and expand Table 1 to present data for each implementation outcome for each country.**

Response: Thank you for the suggestion. We have revised Table 1 to separate the outcomes of acceptability and appropriateness.

As mentioned in the response to comment #2, the majority of the study was conducted in China, therefore, the aim of this study was to summarize findings of implementation outcomes to compare between China and other countries. However, the implementation outcomes by each country were reported in the supplementary: Table S2 for reach and Figure S2 and S3 for fidelity and dose.

We conducted the focus group discussion with the national leaders to better understand the country context, as described in the Methods: “Focus group discussions were conducted by the experienced researcher at the international coordinating center, with site principal investigators (PIs) and national leaders in South America, Nigeria, Pakistan and Vietnam, prior to in-depth interviews to discuss roles and responsibilities, research experience, and how the intervention was implemented at an organizational level.” To avoid duplication of using the quotes, we summarized the contextual barriers across the countries in the Supplemental Table S7.

**6. Table 2 is a similar situation; it should present country-specific information. I think it is fine to have a general summary/explanation for each NPT domain, but without the granularity of each country the paper loses some of its appeal.**

Response: Thanks for the comments. We included the country specific information on the integration of the care bundle into routine care by normalization process theory (NPT) domains, with illustrative quotes in Supplemental Table S6.

**7. Table 3 could be retrustructed to align with the domains and/or the contracts of the Consolidated Framework for Implementation Research (CFIR): Damschroder, L.J., Reardon, C.M., Widerquist, M.A.O. et al. The updated Consolidated Framework for Implementation Research based on user feedback. Implementation Sci 17, 75 (2022).** [**https://doi.org/10.1186/s13012-022-01245-0**](https://doi.org/10.1186/s13012-022-01245-0)

Response: Thanks for the suggestion. In Methods, we added: “To better summarize the contextual factors that identified influence the care bundle implementation, we used Consolidated Framtework for Implementation Research (CFIR) to report”. Table 3 is restructured according to the CFIR framework considering the domains at inner setting, outer setting and individual level:

| **CFIR Domain and construct** | **Contextual barriers** | **Quote examples** |
| --- | --- | --- |
| Outer setting: local conditions | Health care system related financial issues | *“There is also an issue of affordability between private and government hospitals because, however, we do recruit them initially, but their monitoring is sometimes not possible because they have to go to another hospital for their complete management.” (Pakistan, Neurologist)* |
| Outer setting: local conditions and financing | High cost of intravenous antihypertensive agents | *“The intravenous antihypertensive agent alone, is very high cost, so it’s not available for all the patients.” (Focus group, Chile)*  *“Family’s financial is a big issue, some have affordability issue to use medication.” (Pakistan, Neurologist)* |
| Inner setting: work infrastructure | Staffing shortages | *“We had a major disruption and it prevented us from recruiting patients for more than two months because our resident doctors were on strike. During that period, we couldn't get patients to recruit.” (Nigeria, focus group)*  *“We did have some problems with the nursing staff that sometimes - related to glycemic control.” (Mexico, Neurologist)* |
| Inner setting: available resources | Medication limitation | *“With reference to the reversal of anticoagulation- the last treatment target, there is a big difficulty when FFP and PPC are not always available in the hospital” (Vietnam, Neurologist)*  *“We have limited access to intravenous drugs for blood pressure control and that in some cases, made it difficult for us to reach the blood pressure goal.”(Mexico, Neurologist)* |
| Individual: Recipients | Late admission due to lack of community awareness | *“At the patient level, we have significant delays in getting to the hospital on the part of the patients… we had to say that the protocol for Nigeria should be amended so that we have our patients presenting within 24 hours instead of 6 hours that is applied elsewhere.” (Nigeria, focus group)* |
| Inner setting: work infrastructure | Difficulties in the process of information consent | *“An important thing is a false idea that there is still in certain medical teams that cerebral hemorrhage is an issue only in neurosurgical, and that neglects medical care” (Mexico, Neurologist)* |
| Outer setting: local attitudes | Culture and beliefs in clinicians and patients | *“Most of our patients when they have a stroke instead of bringing them to the hospital some will go to the herbalist, our community because they do not believe it is God’s wish, they will believe it is witch or wizard, so they won’t come to the hospital immediately.” (Nigeria, nurse)* |
| Inner setting: work infrastructure; available resources | Limited staffing resources and care space due to COVID-19 pandemic impact | *“Actually, the pandemic is the biggest barrier as mentioned above at affected the recruitemnt and the implementation. there is a shortage of human resources in the department due to some doctors and nurses going to support COVID treatment” (Vietnam, Neurologist )*  *“During the pandemic, ED became the classification department with a high workload, not all the staff could support with a novel procedure. So that many eligible patients were missed because of not adhering to the protocol in some first hours in ED (Vietnam, Neurologist)* |
| Outer setting: local attitudes  Individual: Recipients | Patients/family fear to go to hospitals due to pandemic | *“When the pandemic, the fear appeared… and the hospital policies influence limited the recruitment of patients, and the resource is limited.” (Peru, Focus group)* |
| Inner setting: work infrastructure | Delayed process and limited monitoring due to awaiting PCR results | *“But in the intervention phase, the pandemic affected a lot. For example, subject was admitted but stayed in ED for about 6-8 hours until we have the PCR test confirmed, neurologist only came to access once after the rapid test was negative. It’s hard to ask for operation from ED staff since their workloads were high.” (Vietnam, Neurologist)* |

**8. The discussed is written nicely. It would be relevant to discuss in more detail similarities and discrepancies across countries in terms of implementation outcomes, mechanisms of integration and contextual factors.**

Response: As mentioned in the responses in comment #5, the aim of this study is to compare the implementation of the care bundle between China and other LMICs, this has been discussed in the first paragraph of the discussion. We added discussion of the similarities and differences across the countries on the contextual barriers of the care bundle implementation: “Medication limitation was a common barrier to deliver the care bundle, mainly due to high cost of the medication and insufficient supply at the hospital.” We noted the culture and belief were a specific barrier in Nigeria, which is discussed:” Besides the sociocultural beliefs that impede seeking treatment in acute medical care facilities in Nigeria, the scarcity of culturally appropriate materials about stroke symptoms, and underutilization of ambulance services for stroke patients are known barriers to effective access to stroke services in LMIC.^23,24^ Therefore, further ensuring the Care Bundle is maximally effective requires education of the public about stroke symptoms and timely admission to the hospital to increase the utilization of stroke services in LMICs.”

Yours sincerely,

Menglu Ouyang
