# Supplementary material for: Implementation of a goal-directed Care Bundle for intracerebral hemorrhage: Results of embedded process evaluation in the INTERACT3 trial
Source: PLOS Glob Public Health. 2024 Dec 19;4(12):e0003711. doi: 10.1371/journal.pgph.0003711 (PMC11658503; doi:10.1371/journal.pgph.0003711)
Supplement: Attachment — Submitted filename: Responses to Reviewer_PLOS GPH_final.docx [file pgph.0003711.s015.docx]

6 November 2024

Editorial Office

PLOS Global Public Health

Dear Dr Buna Bhandari,

**Re: PGPH-D-24-01251– “Implementation of a goal-directed care bundle for intracerebral hemorrhage: results of embedded process evaluation in the INTERACT3 trial”**

**We appreciate the comments and suggestions from Editors and Reviewers. We have responded to each of these issues point by point, as outlined below, and made highlighted changes in a revision of the manuscript.**

**Editor Comments:

Thank you for addressing most of the comments in the revised manuscript. However, following questions raised previously has not been addressed. Please address the following concerns along with the reviewer's comments in your revision:

1. In the methods section, three focus group discussions are mentioned as a data collection method, but details about the FGDs are missing, such as how many participants were in each FGD and where (which country) they were conducted. Please provide these details.**

Response: Thanks for pointing this out. The details of the FGDs were added to the Methods: “Focus group discussions were conducted with site principal investigators (PIs) and national leaders in South America (2 PIs), Nigeria (4 PIs), Pakistan (2 PIs) and Vietnam (3 PIs) through the teleconference/video conference, prior to in-depth interviews to discuss roles and responsibilities, research experience, and how the intervention was implemented at an organizational level. Each of the focus group discussions was 30-40 minutes with audio recorded and facilitated by an experienced researcher at the international coordinating centre.” **2. Secondly, although a separate publication from the same study site in China is mentioned in the paper, it is still unclear why the findings from all the countries were not presented together. How do the methods and findings of this paper differ from the study conducted in China, given that it is the same trial site? Additionally, how is a separate publication justified without issues of data slicing? Please clarify your approach.**

Response: Thank you for this clarification. The INTERACT3 trial was first conducted in China in 2017 utilising funding only available for China. We designed the process evaluation (PE) to be conducted at an early phase so that the findings can help to optimise implementation across diverse settings as well as better understand the relationship between elements of the care bundle and outcomes. The research team was then successful for additional funding which was received from UK MRC in 2019 to increase the reach of the trial to other countries.

In China, the process evaluation was conducted between June 2019 to October 2020 and the findings were fed back to the project teams, which informed the implementation across diverse settings. Given the depth and breadth of the PE findings from China purposively sampled across nine sites with data saturation reached, a decision was made to submit the findings from the process evaluation done in China for publication in August 2021. The findings from the China informed the implementation of the intervention in other countries, such as the importance of understanding local health systems prior to the rollout out the study and the need for additional training with the regional coordinating centres about the PE aims and data collection. This has been reported as a reflection in the protocol paper (<https://doi.org/10.3389/fmed.2022.813749>). The data collection of PE in other countries commenced in September 2021.

We decided to report the findings from China and the other countries separately because the PE findings of China sites were submitted in August 2021, which was prior to the PE data collection in the other countries that commenced in September 2021. In addition, considering different health systems and timing of data collection that might be affected by the COVID-19 (data collection in China started pre-COVID while the other countries were post-COVID). We revised the Methods: “Since the trial was initiated earlier in China in December 2017 than in other countries, and the process evaluation for the sites in China was submitted before the study conduction in other countries, this study will mainly focus on the process evaluation in other countries and compare their findings to those findings from China.”

We used consistent methods for the PEs (protocol published <https://doi.org/10.3389/fmed.2022.813749>) and the comparison of the findings has been discussed in the manuscript:“ In comparison to our findings of the PE in China^12^, this study has highlighted the significant adverse impact of the COVID-19 pandemic in other countries on the Care Bundle implementation due to slow recruitment, prolonged consent process and shortage of staffing. Findings in China indicate that BP lowering is already part of routine ICH management but with a wide range of SBP targets applied in the first 24 hours. In contrast, we found there were no standard protocols for BP management for routine care in other countries compared to China. This explains why the differences in BP control between the Care Bundle and usual care groups were larger and the effect of the Care Bundle was greater, in countries outside of China.^7^ Though concerns about the potential harms of intensive BP lowering and challenges to reaching the physiological targets were common in China and other LMICs, the interviewees from outside of China emphasized that sufficient and constant training was essential to support the persistent adoption of the Care Bundle into routine practice.”

**Reviewer #2: Thank you to the authors for revising the manuscript. I appreciate the enhancements made to the manuscript to address my comments. However, I still have a number of points that I believe should be addressed.

1. Although the authors have added more information to the introduction, it still does not fully establish the context and rationale for the specific interventions used in the care bundle. There should be a clearer link between the choice of components in the bundle and their specific relevance to the pathophysiology of ICH in LMICs. For example, more details on the existing standard of care in the included countries and how the care bundle adds value would help strengthen the justification for the study.**

Response: Thanks for the suggestion. We revised the Introduction to provide more rationale of the care bundle components: “Intracerebral hemorrhage (ICH) is the most serious and least treatable form of stroke, accounting for approximately 20% of the 20 million new strokes that occur in the world each year.^1^ However, the burden of ICH is the greatest in low- and middle-income countries (LMIC) where the incidence is very high due to the high prevalence of hypertension, unhealthy diets and other risk factors,^1^ and there is limited access to surgical or medical treatments.^2^ Hematoma expansion (HE) is a strong predictor of disability and mortality after ICH, with every increase of 1 ml in absolute haematoma volume, it leads to a 7% greater likelihood for patients to shift from independence to disability.^3^ HE occurs mostly within 3 hrs from the time of ICH onset and stops progressing after 24 hrs.^4^ Therefore, timely control of HE is an important therapeutic target in ICH management. For many years, clinical trials that focus on a single intervention to control HE have failed to establish a specific beneficial treatment after ICH. This has led to therapeutic nihilism in the approach to ICH care.

Since blood pressure, glycaemia and temperature are often altered from impaired autoregulation after ICH and are associated with haematoma expansion, death and disability, the active management for ICH should target early management of these physiological parameters. A bundled care approach with a set of evidence-informed interventions that are promising to improve patient outcomes. The Australian Quality in Acute Stroke Care (QASC) trial was the first to show that a nursing protocol for the management of fever, hyperglycaemia, and poor swallow improved outcomes for patients with mixed types of stroke. The single-centre before-after Acute Bundle of Care for Intracerebral Haemorrhage (ABC-ICH) found that combined interventions including anticoagulation reversal and BP control, and immediate access to neurosurgical and critical care services reduced 10.8% of the case fatality at 30 days.^5^ However, the previous studies were conducted in Australia and UK, with no evidence of the effectiveness and implementation of the bundled care approach management for ICH in LMICs which has a higher incidence of ICH and different health system contexts. Considering the limited resources such as a lack of neurosurgical settings and speech pathology expertise for dysphagia/swallowing screening in the LMICs, we focused on the management of physiological parameters in our third Intensive Care Bundle with Blood Pressure Reduction in Acute Cerebral Hemorrhage Trial (INTERACT3).” **2. While I understand the logistical and design challenges of conducting a multi-country evaluation, simply grouping the findings from all non-China LMIC sites together may obscure critical contextual differences. For a process evaluation, understanding the local implementation context is crucial. I suggest that the authors provide a more nuanced comparison, highlighting specific variations in implementation across the countries (e.g., differences in healthcare infrastructure, sociocultural factors, or policy environments).**

Response: Thanks for your suggestion. Based on the variations of the contextual factors that have been summarized in Table 3, we added a statement in the Results: “In Latin America, the health system and infrastructure-related challenges impacted the fidelity and dose of the care bundle implementation: there was a lower proportion of patients reached SBP<140 mmHg compared to the overall average (92% vs. 97%) (Figure S3 vs. Table S4) due to the high cost and low availability of the antihypertensive agents; and shortage of the nursing staff resources to monitoring the BGL resulted only 13% of patients reaching the BGL targets (Figure S3). The sociocultural factor of patients’ health seeking behaviour influenced the reach and recruitment of the eligible participants in India and Nigeria. For example, in Nigeria, all the excluded patients were due to delayed admission to the hospital (Table S3) which related to the poor community awareness of stroke symptoms, and beliefs in witchcraft or herbal therapy meant that patients/their families preferred these avenues for treatment rather than seeking immediate attention at a hospital. In Vietnam, the lack of medications to deliver the care bundle had impacted the implementation outcome of fidelity. In Pakistan, health system related factors such as high out-of-pocket costs of treatment and shortage of staffing resources had impacted the fidelity of the implementation, showing a lower proportion of patients received treatment for glucose control (20%) and pyrexia (56%) compared to the other countries (Figure S2). The insurance and the financial burden of the treatment also influenced the uptake of the treatment by patients: there were 27% of the participants refused to give consent to participate the study (Table S3). In Latin America, Sri Lanka and Vietnam, healthcare infrastructure factors such as delays in the process of diagnosis and limited resourcing (e.g. staff, space) during the COVID pandemic were the main barriers to recruiting eligible patients and delivering the care bundle, which impacted upon the implementation outcomes of reach and fidelity. For example, there were a lower percentage of participants in Chile/Peru, and for those excluded patients in Peru were mainly due to delayed admission (>6 hrs from stroke onset) compared to overall average (85.2% vs. 67.5%). A summary of the contextual barriers that impeded the care bundle implementation by country is reported in Table 3.”

**Table 3. Contextual factors according to participating countries**

| **Country** | **CFIR Domain and construct** | **Contextual barriers** | **Illustrative quotes** | **Implementation outcomes impacted upon** |
| --- | --- | --- | --- | --- |
| Chile/Peru/Mexico | Outer setting: local conditions- Healthcare system related financial | High cost of the medication | *“The intravenous antihypertensive agent alone, is very high cost, so it’s not available for all the patients.” (Focus group, Chile)* | Fidelity |
|  | Inner setting: available resources | Limited accessibility of the medication | *“We have limited access to intravenous drugs for blood pressure control…made it difficult for us to reach the blood pressure goal.”(Mexico, Neurologist)* | Dose |
|  | Individual: Recipients-Sociocultural | Pandemic resulted in late presentation due to patients/families fear of going to hospitals | *“When the pandemic, the fear appeared in patients… and the hospital policies influence limited the recruitment, and the resource is limited.” (Peru, Focus group)* | Reach |
|  | Inner setting: healthcare infrastructure | Shortage of workforce | *“We did have some problems with the nursing staff that sometimes - related to glycaemic control.” (Mexico, Neurologist)* | Dose |
| Nigeria | Inner setting: available resources | Limited resources for diagnosis | *“I think some hospitals might not have the neuro-imaging techniques necessary to make the diagnosis.” (Nigeria, Neurologist)* | Reach |
|  | Inner setting: available resources | Short of staff resourcing due to hospital strike during COVID pandemic | *“We had a major disruption and it prevented us from recruiting patients for more than two months because our resident doctors were on strike. During that period, we couldn't get patients to recruit.” (Nigeria, focus group)* | Reach |
|  | Individual: Recipients-Sociocultural | Late admission due to lack of community awareness of stroke and culture/beliefs of patients about seeking treatment of stroke | *“Most of our patients will go to the herbalist, our community because they do not believe it is God’s wish, they will believe it is witch or wizard, so they won’t come to the hospital immediately.” (Nigeria, nurse)*  *“At the patient level, we have significant delays in getting to the hospital on the part of the patients….” (Nigeria, focus group)* | Reach |
| Vietnam | Inner setting: work infrastructure | Pandemic caused workforce shortage and a delayed process in diagnosis | *“The pandemic is the biggest barrier affected the recruitment and the implementation...there is a shortage of human resources in the department” (Vietnam, Neurologist)*  *“Subject was admitted but stayed in ED for about 6-8 hours until the PCR test confirmed…It’s hard to ask for operation from ED staff since their workloads were high.” (Vietnam, Neurologist)* | Reach |
|  | Inner setting: available resources | Medication limitation for care bundle delivery | *“With the reversal of anticoagulation, there is a big difficult when FFP and PPC are not always available in the hospital” (Vietnam, Neurologist)*  *“Hospitals that do not have nicardipine or equipment or drugs to manage blood glucose…room conditions might not be adequate.” (Vietnam, Neurologist)* | Fidelity |
| Pakistan | Outer setting: local conditions- Health care system related financial | Health care system related financial issues due to insurance cover | *“There is also an issue of affordability between private and government hospitals…their monitoring is sometimes not possible because they have to go to another hospital for their complete management.” (Pakistan, Neurologist)*  *“Family’s financial is a big issue, some have affordability issue to use medication.” (Pakistan, Neurologist)* | Reach and Fidelity |
|  | Inner setting: available resources | Work overload | *“Other issues are staff resources related, as discussed earlier they are busy, and they are overloaded as a result” (Pakistan, Nurse)* | Fidelity |
| Sri Lanka | Inner setting: available resources | Unavailable to report INR due to lack of lab resources | *INR was difficult due to unavailability of reports as early as we would like it to be ready” (Sri Lanka, Neurologist)* | Dose |
|  | Individual: Recipients - sociocultural | Pandemic caused workforce shortage | *“Due to the COVID pandemic, our ward staff and space facility is limited, such as the availability of high dependency units and monitoring.” (Sri Lanka, Neurologist)* | Reach |
| India | Individual: Recipients - sociocultural | Delayed admission to hospitals due to patients’ health seeking behaviour to seek treatment from local doctors | *“We should increase awareness of stroke in the community first … to receive the stroke patients without delay.” (India_002)*  *“Recruiting patients was difficult because most of the patients go to local doctors and there’s a lot of delay in the tertiary care hospital” (India, Neurologist)* | Reach |

CFIR denotes Consolidated Framework for Implementation Research, ED emergency department, FFP fresh frozen plasma, PPC prothrombin complex concentrate

**3. The use of the term “vanguard phase” to describe the separate process evaluation for China is still a bit ambiguous. It would be helpful if the authors clearly articulate the purpose of this phase, its specific objectives, and why the decision was made to analyze these data separately. Additionally, they should discuss how findings from the China phase were used to inform implementation in the subsequent countries.**

Response: Thank you, please see response to the editor’s second comment above which seeks to clarify our approach for the early phase which was to use the PE findings in the early phase to optimise trial implementation, and our decision to analyse and report these data separately. Additionally, we have made edits to be more explicit in how the findings from the China phase informed the implementation in subsequent countries.

**4. The authors have added some country-specific information in supplementary tables, but these are not integrated into the main text, making it difficult to interpret the contextual nuances of implementation across countries. A more thorough country-specific comparison within the main text would add significant value to the paper and support the argument that implementation is context-dependent.**

Response: Thank you for your suggestion. We have integrated the country specific information in the main text and also included examples in Table 3. Please see the responses to the comment #2.

**5. The restructuring of Table 3 based on the CFIR framework is a step in the right direction. However, the information remains too general. Each CFIR domain should have clearer links to how specific contextual factors influenced implementation success or failure in each country. Additionally, illustrative quotes should be tied more directly to the relevant constructs to make the table more informative.**
Response: Thanks for the comments. The specific contextual factors that were identified as barriers to implementation outcomes of the Care Bundle in each country have been summarized in Table 3, with some context-specific country examples included in the main text, please see the responses to the comment #2.

In addition, we have chosen the most representative illustrative quotes for the CFIR domain and its impact on the implementation outcome.

**I believe addressing these points would add value to the manuscript and render it suitable for publication.**

Response: Sincere thanks for your comments and suggestions, which have helped to improve the manuscript a lot.
